# A Field Calibration Solution to Achieve High-Grade-Level Performance for Low-Cost Dual-Frequency GNSS Receiver and Antennas

**DOI:** 10.3390/s22062267

**Published:** 2022-03-15

**Authors:** Andreas Krietemeyer, Hans van der Marel, Nick van de Giesen, Marie-Claire ten Veldhuis

**Affiliations:** 1 Faculty of Civil Engineering, Delft University of Technology, 2628 CN Delft, The Netherlands; h.vandermarel@tudelft.nl (H.v.d.M.); n.c.vandegiesen@tudelft.nl (N.v.d.G.); j.a.e.tenveldhuis@tudelft.nl (M.-C.t.V.); 2 R&D Department of Seismology and Acoustics, Royal Netherlands Meteorological Institute (KNMI), Utrechtseweg 297, 3731 GA De Bilt, The Netherlands

**Keywords:** GNSS, receiver antenna calibration, Phase Center Variation (PCV), ZTD, positioning, U-Blox, ZED-F9P, low-cost

## Abstract

Low-cost dual-frequency receivers and antennas have created opportunities for a wide range of new applications, in regions and disciplines where traditional GNSS equipment is unaffordable. However, the major drawback of using low-cost antenna equipment is that antenna phase patterns are typically poorly defined. Therefore, the noise in tropospheric zenith delay and coordinate time series is increased and systematic errors may occur. Here, we present a field calibration method that fully relies on low-cost solutions. It does not require costly software, uses low-cost equipment (~500 Euros), requires limited specialist expertise, and takes complex processing steps into the cloud. The application is more than just a relative antenna calibration: it is also a means to assess the quality and performance of the antenna, whether this is at a calibration site or directly in the field. We cover PCV calibrations, important for deformation monitoring, GNSS meteorology and positioning, and the computation of PCOs when the absolute position is of interest. The method is made available as an online web service. The performance of the calibration method is presented for a range of antennas of different quality and price in combination with a low-cost dual-frequency receiver. Carrier phase residuals of the low-cost antennas are reduced by 11–34% on $L_{1}$ and 19–39% on $L_{2}$, depending on the antenna type and ground plane used. For the cheapest antenna, when using a circular ground plane, the $L_{1}$ residual is reduced from 3.85 mm before to 3.41 mm after calibration, and for $L_{2}$ from 5.34 mm to 4.3 mm. The calibration reduces the Median Absolute Deviations (MADs) of the low-cost antennas in the vertical direction using Post Processed Kinematic (PPK) by 20–24%. For the cheapest antenna, the MAD is reduced from 5.6 to 3.8 mm, comparable to a geodetic-grade antenna (3.5 mm MAD). The calibration also has a positive impact on the Precise Point Positioning (PPP) results, delivering more precise results and reducing height biases.

## 1. Introduction

**Figure 1:**
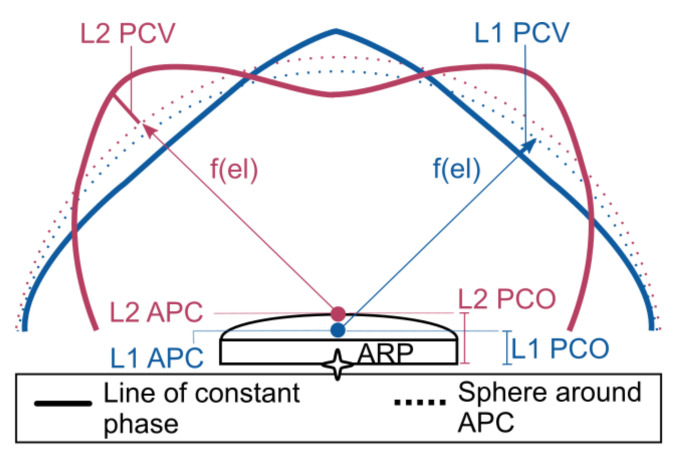
Schematic illustration of Antenna Reference Point (ARP, black star symbol), Antenna Phase Center (APC, filled circles), lines of constant phase (solid colored lines), and spheres around APC points (dashed lines) on $L_{1}$ (blue) and $L_{2}$ (red). The Phase Center Variations (PCVs, thick colored lines between the spheres around APC and line of constant phase) are shown as functions of the elevation f(el) on the respective frequency.

The antenna is a crucial part of the Global Navigation Satellite System (GNSS) ground receiver setup. It is responsible for receiving the signals transmitted by GNSS satellites, transforming the electromagnetic energy into electric currents, and forwarding it to the GNSS receiver. In an ideal scenario, the phase pattern of an antenna is constant, which introduces the same phase delay or advance in all directions. In reality, this phase pattern is irregular and depends on the antenna characteristics, environment, and receiving signal frequency [1]. The estimated satellite range then refers to an imaginary phase center. This imaginary phase center is actually not a single point, or a point at all, as it depends on the frequency of the signal and line of sight to the satellite. To relate this phase center to a physical reference point on the antenna, the Antenna Reference Point (ARP), two steps are used. The first part is a correction in the line of sight to an intermediate point, called the Antenna Phase Center (APC). This is the so-called Phase Center Variation (PCV). The PCV is the projection onto the line of sight of the difference between the actual phase center and the APC. It is therefore a correction based on the elevation and azimuth angle between the actual phase center and the satellite. Representing this correction, the PCV is usually given as a table in elevation and azimuth angle, with different tables for each frequency. The second part, called the Phase Center Offset (PCO), is the offset between the ARP and APC. This is illustrated in Figure 1. The ARP is typically defined as the intersection of the vertical symmetry axis with the bottom of the antenna [2], which is a clearly identifiable physical point on the antenna. The definition of the APC is more open. It is common practice to use a different APC for each frequency, though it is also possible to use a single APC for all frequencies. It is also common practice to choose the APC such that the PCVs become small numbers (as far as this is possible). The PCV and PCO are related: change one, and you should change the other. A good operational practice is to define the APC as the point for which the mean of the PCVs is zero. Since the PCVs are typically different for each frequency, setting the mean of the PCVs to zero would lead to a different APC for each frequency. This can be considered as a Mean Phase Center. The advantage of this definition is that users may choose to ignore the PCV correction, and only use the larger PCO correction. The magnitude of the PCV typically ranges from several mm to a few cm [3]. The PCO can be several centimeters in the vertical direction, and a few mm in the horizontal direction. The problem with the mean phase center definition of the APC is that, apart from the antenna characteristics and environmental factors (multipath), also the cutoff angle and processing strategy (weighting of observations) influence the APC definition [4]. In this paper, we do not always follow the mean phase center definition for the APC. We see the APC more as an intermediate point, that often has no practical value at all. For GNSS meteorology, the absolute APC, and thus the absolute PCO, has no practical value, only the differential (between frequency) PCO matters. Unlike positioning, for GNSS meteorology, it is more important to apply the PCV than the absolute PCO corrections. The same is true for deformation monitoring as long as we do not change instruments. The standard format for PCVs and PCOs is the IGS Antenna Exchange Format (ANTEX).

To specify antenna characteristics, individual antennas need to be calibrated. One distinguishes between relative calibrations obtained from a base–rover setup and absolute calibrations obtained in the field with a robotic arm [5,6,7] or in an anechoic chamber [8] using artificial GNSS signals. The antenna, but also the utilized ground plane, can influence the phase characteristics. Tests with different ground planes were conducted, e.g., by [9] or [10]. By combining multiple individual calibrations of the same antenna model, type-mean calibrations can be generated. These calibrations are publicly available for an increasing number of geodetic antennas, e.g., by the International GNSS Service (IGS; [11]). With the advent of low-cost receiver and antenna setups for positioning or tropospheric delay analysis, there is a need for the calibration of low-cost antennas. A recent study by [12] found high PCVs of up to 2 cm on L1 and 4 cm on L5 after performing an absolute antenna calibration with a robotic arm using a Huawei Mate20X dual-frequency smartphone. The study by [13] designed a 3D displacement detection test with a u-blox ZED F9P receiver with ANN-MB-00 low-cost antennas and found minimum detectable displacements of 10 mm upwards. Calibrating low-cost antennas is essential to improve their performance, yet individual calibrations are not available for free for such antennas and the calibration, e.g., by a robot, is costly and contradicts the idea of low-cost solutions.

A low-cost solution is needed to fully exploit the potential of high-precision positioning applications using low-cost receiver and antenna setups for users of different disciplines (surveying, mapping of rivers, ground control points, or atmosphere monitoring). This study uses a low-cost dual-frequency receiver (u-blox ZED F9P) together with antennas of different quality in the price range of 50 to 1000+ Euros. A relative antenna calibration [4] is performed. The assumption is that if two antennas with the same antenna phase pattern are used, these effects cancel out over a short baseline. Using different antennas for base and rover results in differences that are reflected in the estimated residuals. The obtained relative calibrations are converted to absolute ones by using a base station antenna with known antenna calibration. The presented antenna calibration tool is made available online under https://gnss-antcal.citg.tudelft.nl (accessed on 9 February 2022). It allows users to calibrate their own antenna in the field, provided that a base antenna with known calibration is utilized. The presented method is relying fully on low-cost solutions. It does not require costly software, uses low-cost equipment (about 500 Euros), and provides an inexpensive solution for calibration. The only necessity is access to an antenna for which the antenna calibration parameters are known (e.g., type-mean calibrations from the IGS ANTEX file), or an already individually calibrated antenna for the base station. This can be achieved either by performing the calibration near an already established base station, e.g., a Continuously Operating Reference Station (CORS), or by renting, borrowing, or purchasing a high-grade calibrated antenna for a few days. This opens a wide range of new application domains, especially since this can be done with little or no expertise because complex processing steps are taken into the cloud. Performance of in-field calibrated low-cost receiver and antenna setups is demonstrated using static Precise Point Positioning (PPP) and Post Processed Kinematic (PPK) processing. The presented solution can be particularly interesting for areas where high-precision positioning is not available or application remains limited due to high costs. The application is not only a relative calibration, but also a means to assess the quality and performance of the antenna, whether this is at a calibration site or in the field. Though the PCO estimation is also covered, the core of this study is on the effectiveness of using only PCVs for, e.g., GNSS meteorology, where the absolute position is not important or of lesser interest. A different calibration strategy (e.g., by robot or in an anechoic chamber) is recommended when the PCOs are the main goal of the calibration.

In the next sections, we describe the experimental setup, the calibration procedure, and its performance on the residuals after calibration. Then, we present the online tool, followed by a discussion of the obtained positioning results. Finally, we draw the conclusions and propose directions for future work.

## 2. Experimental Setup

A series of short baseline experiments, utilizing a u-blox ZED-F9P receiver connected to different quality and type antennas (rover) together with the IGS station DLF1 (base station, IGb14 XYZ[m] coordinates at epoch 001/2010: 3924697.776, 301125.106, 5001905.251), was conducted at the rooftop of the Netherlands Metrology Institute (NMi) in Delft. The rover antennas were installed consecutively on the pillar DOMES 13502M003 (GPS Mark 15), which is 10.404 m East, 6.928 m North, and 1.468 m Down (with a standard deviation of 1–2 mm) from the base station antenna. The installation situation is characterized by an almost unobstructed view over the full horizon and, compared to a real-life environment, the ground multipath caused by reflections of other objects and near-field effects (scattering caused by surfaces in the near field, e.g., the pillar) can be regarded as relatively clean. Nevertheless, these effects will be included in the estimated residuals. With a baseline length of approximately 10 m, delays caused by the troposphere and ionosphere can be regarded as equal for base and rover. These effects are considered as canceled in the differential processing.

The base station consists of a Trimble NetR9 receiver connected to a Leica AR25.R3 (LEIAR) antenna with LEIT radome. The investigated rover antennas are the low-price antenna u-blox ANN-MB-00 (ANN-MB), the medium-priced antennas Trimble AV28 (AV28) and Trimble GA530 (GA530), and the geodetic-quality antennas Trimble TRM55971.00 NONE, also known as Zephyr 2 Geodetic (TRM5), and Leica AR25.R3 LEIT (same model as base station antenna). Because of the ANN-MB (antenna without screw mount) and Trimble AV28 (screw-in hole mount) antenna designs, antenna mounting adaptors were constructed. One is a metallic, rectangular bracket, and the second one is a plastic bracket with a metallic circular disk of 10 cm diameter on top. Since these are two different recording sessions using two different mounting brackets, two different datasets exist for the AV28 and ANN-MB antennas. The information is summarized in Table 1.

The antennas cover a price range from low (<100 Euros) to high cost (>1000 Euros). All rover antennas are connected to a u-blox ZED-F9P receiver. Official IGS ANTEX type mean antenna calibrations exist for the highest-quality rover antennas (LEIAR and TRM5) as references. The LEIAR antenna calibration is used in the calibration step (see Section 3).

The rover and base logging interval is set to 1 Hz. Using a lower frequency, with a 10- or 30-s sample rate, is possible, but will decrease the precision of the estimated PCVs slightly. Base station data are obtained from the Dutch Permanent GNSS Array (DPGA) in 15-min high-rate (1 Hz) RINEX (Receiver Independent Exchange Format) files. These are merged to daily 1 Hz data observation files. Rover data are logged on a local SD card and converted to daily RINEX files for post-processing. Since not all antennas use the same antenna adaptor, the heights from the GPS mark to the antenna ARPs are measured manually. With the exception of the circular adaptor (used by AV28 and ANN-MB), which is off by 10 mm North and 8 mm East, only height offsets are introduced by the antenna adaptor. Though measured with care, the manual measurements have a precision error of approximately 1 mm.

## 3. Calibration Methodology

The goal of our calibration is to estimate elevation-averaged PCVs in ANTEX format for the investigated rover antennas using the residuals obtained using a short baseline. The obtained PCVs can be applied to the rover carrier phase observations to improve the residuals (see Section 4) and positioning results (see Section 6).

With our method, which is a relative antenna calibration, site-dependent factors that are different between base and rover antenna will affect the estimated residuals and will therefore directly influence the estimated PCVs. The site-dependent factors (multipath) are unique for each installation and will influence the estimated parameters. They cannot be covered fully in antenna calibrations except if the calibration is performed in the same spot as the experimental location. Three scenarios are possible: (i) bring the rover to the base (calibration site) for calibration, (ii) bring the base (with geodetic grade calibrated antenna) to the rover (in-situ) for calibration, or (iii) do an in-situ calibration. Only when the calibration is done completely in situ (i.e., the same base is used for calibration and the experiment) site-dependent effects will be mitigated. However, this requires the rover and/or base not to change after the calibration is done. This means processing relatively long baselines during the calibration phase, possibly taking multiple days. Another interesting option is to use Virtual Reference Station (VRS) data. These could be utilized in zero-baseline mode. The second-best option is number (ii), where the base station is temporarily brought to the experiment site. This is inverting scenario (i). It also assumes that the local site effects are smaller when the calibrated geodetic antenna is brought to the rover, instead of bringing the rover to a calibration site (option i). The words “antenna calibration” can be slightly misleading in this context: we have in mind to calibrate low-cost antennas in their final settings as much as possible, including local site effects, and not per se only the antenna itself.

Apart from the residuals, only the time-independent static baseline vector, time-dependent clock, and carrier phase ambiguity parameters are estimated in the calibration step. The data processing is primarily realized using the open-source tool RTKLIB [14]. The applied processing options can be found in Table A1 in the Appendix A. One result of the RTKLIB process is Double Differenced (DD) carrier phase residuals for each frequency and each common satellite with a resolution of 0.1° in elevation and azimuth. To obtain Single Differenced (SD) residuals, which are required to analyze the PCVs, the RTKLIB source code is modified to subtract the average of the DD residuals from the DD residuals. See also Github issue: https://github.com/tomojitakasu/RTKLIB/issues/457 (accessed on 9 February 2022). It avoids calculating the SDs from the DDs manually. However, if users prefer to not modify the code, but apply the DD to SD correction manually, the following formula (after [15]), assuming a zero-mean condition, can be used:

(1) $$SD_{i}=DD_{i}-mean\left(DD_{0\ldots n}\right),$$

with *i* being the satellite at the specified epoch and frequency and *n* being the number of DDs on that frequency and epoch.

We use GPS $L_{1}$ and $L_{2}$ observations exclusively to keep it simple and because most existing antenna calibrations to date (which are used for validation of the method) are available for these frequencies. However, the method can easily be extended to different frequencies and systems. The ambiguity-fixed residuals over three full observation days for all antennas, except ANN-MB with a circular adaptor, which uses only two full days, were selected and stacked over 0.1° elevation and azimuth bins. The days were manually selected based on a visual inspection of the ambiguity-fixed phase residuals. To investigate the effect of different averaging widths, the mean of the stacked residuals was applied to obtain elevation-only averages in the resolutions 0.1°, 1°, and 5°. The elevation-only approach was preferred over an azimuth- and elevation-dependent approach since the observation period was short and no antenna rotation was performed to cover all parts of the antenna. A previously conducted test with both methods also revealed a slight performance decrease in the ZTD estimation [16] using the latter. A figure illustrating the elevation-only phase residuals can be found in the Appendix A.

To apply the antenna corrections, the mean of the stacked SD residuals could be added directly to the rover observations. However, this would limit the application to the analyzed baseline and could not be applied to other locations. To obtain PCVs independent of the analyzed baseline, absolute PCVs of the rover antenna are needed. We achieve this by adding the absolute PCVs from the base station antenna (LEIAR25.R3 LEIT) to the stacked relative residuals between base and rover. We use the base station antenna entry from the IGS type mean ANTEX file (I14.ATX) for this purpose. This creates PCVs for each investigated rover antenna for each frequency. Since three different resolutions (0.1°, 1°, and 5°) are investigated, a new ANTEX file is created for each of them. To further generalize the presented method and since not all processing engines allow us to supply our own ANTEX files to their processing chain, the base and rover RINEX files are manipulated according to the PCVs of their respective antennas. We adjust the phase data using the following formula:

(2) $${\tilde{L}}_{i}=L_{i}-\frac{\phi_{i}^{r}\left(el,az\right)}{\lambda_{i}},$$

with (${\tilde{L}}_{i}$) being the corrected phase data on frequency *i* and $L_{i}$ the original RINEX phase observation in cycles. The azimuth- and elevation-dependent ANTEX PCV correction $\phi_{i}^{r}\left(el,az\right)$ is scaled by the wavelength $\lambda_{i}$.

The following section describes the impact of the calibration step on the estimated residuals. Positioning results can be found in Section 6.

## 4. Calibration Impact on the Estimated Residuals

To investigate the performance of the antennas after applying the corrections to the base and rover RINEX files, another PPK run over the same short baseline is performed using the same observation dates as in the calibration step. The only difference lies in applying the PCVs to the base and rover RINEX carrier phase observations (see Equation (2)). As a metric for the comparison, we use a robust estimator of the standard deviation, the Median Absolute Deviation (MAD):

(3) $$\mathrm{MAD}=1.4826\cdot median(|x_{i}-\tilde{x}|).$$

**Figure 2:**
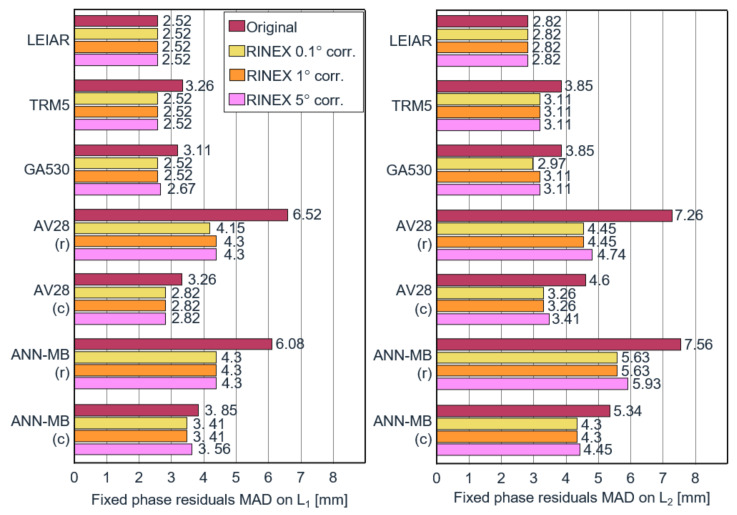
Ambiguity-fixed phase residuals on $L_{1}$ (**left**) and $L_{2}$ (**right**) before and after the calibration using 0.1°, 1°, and 5° binning widths. The abbreviations (c) and (r) in the antenna names correspond to the utilized mounting brackets (circular plane and rectangular bracket).

The variables $x_{i}$ and $\tilde{x}$ depict the observations and the median of the data. The scale factor of 1.4826 is constant and is multiplied by the median of the absolute deviations. The scale relates to the underlying assumption of normally distributed data. Compared to the standard deviation, the MAD is more resistant to outliers and, therefore, our preferred measure in this study. The MADs of the ambiguity fixed phase residuals before and after calibration are illustrated in Figure 2.

The MADs of the ambiguity-fixed phase residuals are generally lower for the more expensive antennas and higher for the lower-cost antennas. The highest-quality antenna (LEIAR) results in the lowest phase residuals (MAD of 2.52 mm on $L_{1}$ and 2.82 mm on $L_{2}$), which also remain unchanged after correcting the input data. It shows that the LEIAR antenna patterns of both base and rover are very similar so PCVs cancel out over a short baseline during the calibration step. Compared to LEIAR, which was also used by the base, the TRM5 and GA530 have before correction an MAD on $L_{1}$ that is 29% and 23% higher, and 36% higher on $L_{2}$. After correction, the MAD on $L_{1}$ is of the same level and just slightly above on $L_{2}$. For the lower-cost antennas AV28 and ANN-MB with a circular ground plane, the MAD on $L_{1}$ before correction is, respectively, 29% and 53% above the reference antenna, and on $L_{2}$, respectively, 63% and 89%. After adjusting the phase data, these numbers are reduced to a difference of 12% and 35% on $L_{1}$ and 16% and 52% on $L_{2}$. The results with a rectangular adaptor demonstrate the highest deviations but also the highest improvements (from around 150% down to 60–100%). Though considerably improved, the performance is clearly below its counterparts utilizing a circular ground plane. Interestingly, the different resolutions of the applied PCV corrections only marginally affect the outcome. Compared to the results without calibrations and depending on the antenna type and ground plane, the residuals of the low-cost antennas were reduced by 11.4–34% on $L_{1}$ and 19.5–38.7% on $L_{2}$.

**Figure 3:**
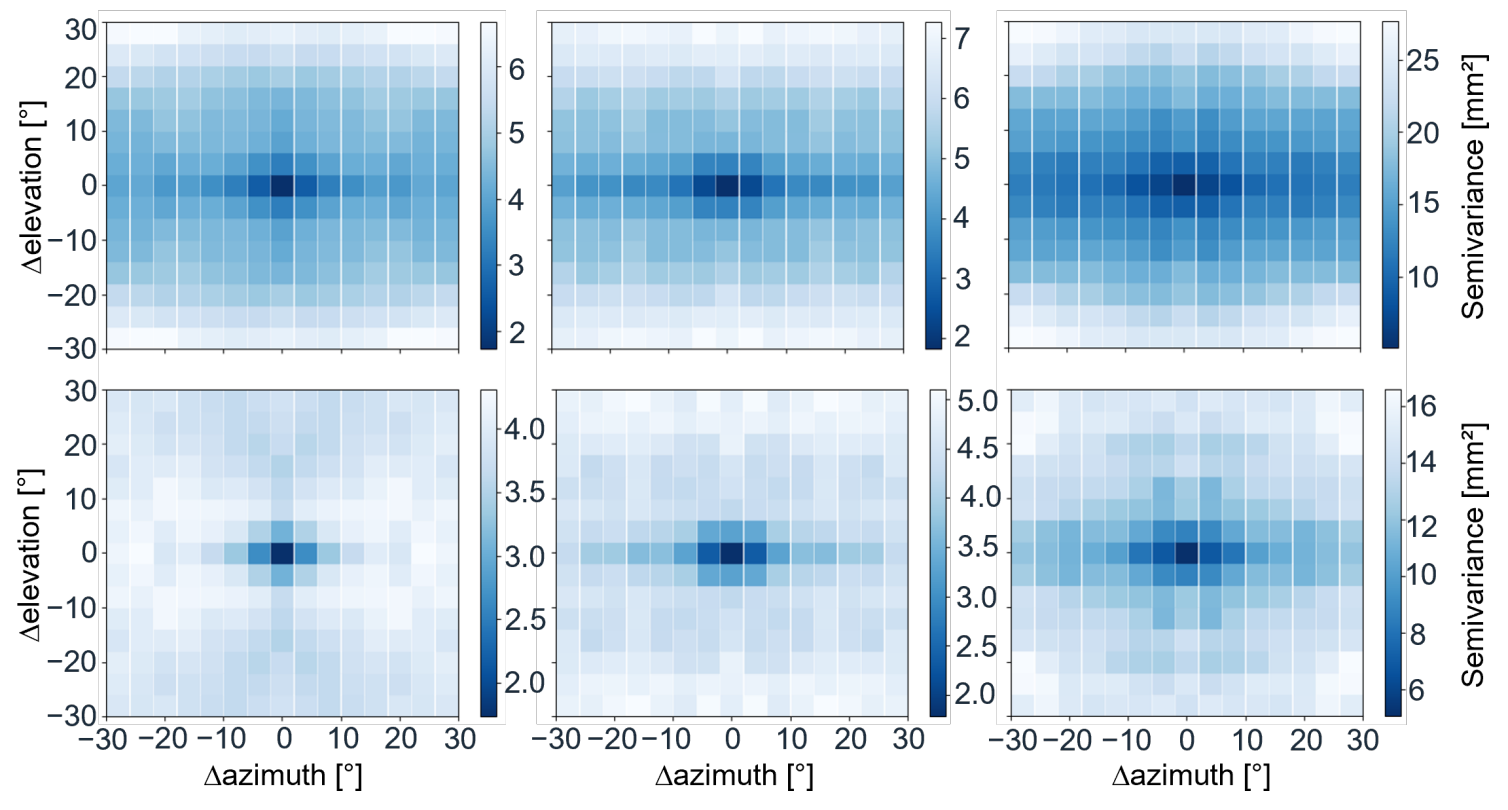
The 2D variogram maps of the residuals for the antennas TRM5 (**left**), AV 28 circular ground plane (**middle**), and AV28 rectangular adaptor (**right**), before (**top**) and after (**bottom**) removing the elevation pattern.

The results suggest that, even after correction, noise is still present in the data, which could not be compensated by the mean of the stacked residuals. The random component of carrier-phase noise and model limitations are related to the finite bin size in azimuth and/or elevation. To further examine the effectiveness of the elevation-only averaging, we calculate the semivariance $\hat{\gamma }$ (see, e.g., [17]) for each antenna before and after calibration over the full elevation and azimuth horizon to determine if only white noise is left in the residuals:

(4) $$\hat{\gamma }=\frac{1}{2N(d)}\sum_{\left|\right|u_{i}-u_{j}\left|\right|}{\left(z\left(u_{i}\right)-z\left(u_{j}\right)\right)}^{2},$$

where N(d) is the number of data pairs belonging to the data bin *d* and *z* are the paired observations at spatial locations u separated by the Euclidean distance $\left|\right|u_{i}-u_{j}\left|\right|$. We conduct the grouping by separating the data points into bins of distinct distances using the 1° resolution averages. Figure 3 shows the 2D variogram maps of the antennas TRM5 and AV28 with a circular and rectangular adaptor using $L_{1}$ observations before and after applying the corrections. Each pixel shows the averaged semivariance over 4° and covers the spatial variance over up to 30° azimuth and 30° elevation difference.

All antennas have in common that the semivariance generally decreases after removing the elevation pattern. However, the residuals are still correlated (low semivariance) over short distances, independent of removing the antenna pattern. This is an indication that remaining errors are not just white noise. After removal of the elevation pattern (calibration), the semivariance for the TRM5 antenna is the smallest, with reduced correlation in both the elevation and azimuth direction, especially in elevation. This is followed by the AV28 antenna with the circular ground plane. For the AV28 antenna, with a rectangular adaptor, azimuth- and elevation-specific artifacts remain that could only partially be covered by the calibration. The semivariogram maps of all antennas can be found in the Appendix A.

## 5. Online Web Service

The presented calibration method is implemented as an online web tool (https://gnss-antcal.citg.tudelft.nl, accessed on 9 February 2022). It calculates the baseline between the base and rover antenna and provides the estimated PCVs of the rover antenna for GPS $L_{1}$ and $L_{2}$. An antenna calibration can therefore be directly performed by the user in the field.

**Figure 4:**
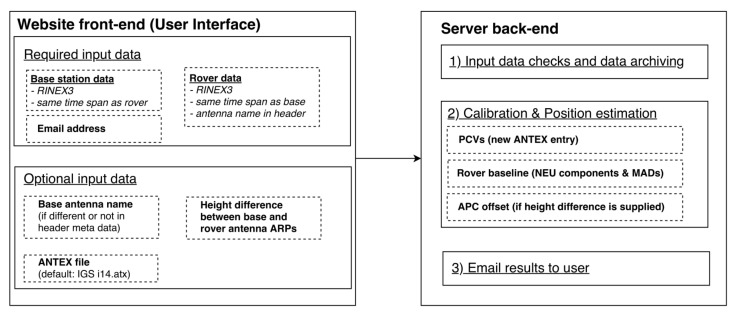
Overview of the web tool requirements and functions.

To calibrate a rover antenna with the online calibration tool, a simple base–rover setup is required. The base station antenna must be an already calibrated antenna with available calibration patterns in the current type mean IGS ANTEX file or in an external ANTEX file. For the best results, both antennas are placed over a very short baseline and the rover antenna is installed at the location as it is intended to stay after the calibration. In any case, both antennas should be installed in a preferably multipath-free environment with no or only small height differences between them. The tool in its current state only accepts RINEX3 files that cover a maximum of one full day. The user is advised to format the input data accordingly to avoid rejection of the input data by the software. On the website, an explanation page highlighting the requirements is provided. The basic specifications and features of the tool are illustrated in Figure 4.

The user can provide the base antenna name separately or specify it in the RINEX header. If no custom ANTEX file is supplied, the currently available IGS ANTEX I14.ATX is used. The name of the base antenna must be in the ANTEX file. The user is also required to supply the rover RINEX header with an antenna name. The recommended maximum sampling frequency should not exceed 1 Hz. The user is encouraged to upload GPS-only observations with $L_{1}$ and $L_{2}$ observations as these are the only frequencies that are currently supported by the tool. However, multi-GNSS observations can also be uploaded but will not be utilized by the current processing scheme. The program will output the computed baseline between base and rover antenna in local coordinates (North, East, and Up), as well as an ANTEX entry containing the PCVs. To be consistent with the ANTEX notation, an averaging of 5° is chosen. If the height difference between the ARP of the base antenna and the ARP of the rover antenna is provided by the user (mm level), the estimated median height offset will be reported as PCO in the ANTEX entry on both supported frequencies.

## 6. Positioning Analysis

To evaluate the performance of the calibration method, this section analyzes the positioning results from the investigated antennas using static PPP and kinematic PPK, with and without applying the antenna calibrations. The main focus is set to the height estimation. Results for the horizontal components can be found in the Appendix A. As mentioned before, only GPS $L_{1}$ and $L_{2}$ observations are used. The high-quality LEIAR25.R3 LEIT antenna serves as a reference for our analysis. The same model is also used as a base station antenna, which eliminates antenna-induced effects in the differential PPK processing. Unless mentioned otherwise, 1° resolution elevation antenna phase corrections are used.

### 6.1. NRCan PPP Results

With the online service NRCan (National Resources Canada) PPP [18], the ionosphere-free linear combination of $L_{1}$ and $L_{2}$ phase observations ($L_{IF}$) is applied. The resulting mean phase center is consequently adjusted to the linear combination of the $L_{1}$ and $L_{2}$ phase centers. This experiment allows us to estimate the $L_{IF}$ PCO and the impact of the PCVs on the height estimations. The NRCan service uses IGS products and applies the type mean antenna phase center corrections from the current IGS ANTEX file. The estimated height of the rover LEIAR antenna, with antenna corrections from the IGS ANTEX file applied by the PPP engine, is used as the reference value for the height. Moreover, for the TRM5 antenna, which has an entry in the IGS ANTEX file, the PPP engine applies antenna corrections automatically. This antenna serves as an additional reference.

The PPP analysis is done by submitting several dual-frequency RINEX files of one full day. Since the high-rate (1 Hz) data sampling rate is not required for the static PPP processing, the data are down-sampled to a 30-s interval. Ocean loading parameters for the station location from the FES2004 model [19] are provided in an external file. Final GPS orbit and clock parameters are used by the engine. The antenna corrections (PCO and PCVs) are automatically applied to the receiver antenna provided that the antenna name is given in the RINEX header and an entry for this antenna (and radome) exists in the IGS ANTEX file. The engine also applies the satellite antenna corrections. Except for the input RINEX data, all conducted tests use the same configuration parameters.

To verify the implementation of the corrections to the RINEX data, the reference antennas LEIAR and TRM5 are processed by the PPP engine using different input data configurations. The results can be found in Table 2.

The first line shows the offsets using the original RINEX data without antenna metadata. No receiver antenna correction is applied, therefore yielding the position of the mean phase centers of the $L_{IF}$ of the combined PCO and PCV effects. The resulting offsets are 183 and 71 mm. The second line shows the height differences with automatically applied IGS PCO and PCV corrections. Obviously, no height error is found for the LEIAR antenna since it serves as our reference measurement. A difference of −8 mm compared to the LEIAR ARP position is observed for the second antenna. Though the same mounting adaptor was used for both antennas, this antenna could not be fully screwed into the screw hole and the height offset was measured manually and corrected by 10 mm. This is already taken into account in the values given in the table. The higher uncertainty of the manually measured height could play a role in this observed offset of −8 mm, but another, more likely reason is that, because the LEIAR and TRM5 were mounted on different days, the difference is simply due to noise in the data. The third line shows the height differences after manually applying the IGS ANTEX PCV (but no PCO) corrections to the RINEX files according to Equation (2). The RINEX modification is necessary since it is not possible to supply your own ANTEX file to the NRCan online service. To be consistent with the IGS ANTEX resolution, this analysis is performed using an angular resolution of 5°. The implemented interpolation strategy in the NRCan software is unknown to us. For our cases, we use linear interpolation to the respective satellites to obtain the phase corrections. The resulting offsets respect the PCVs and return an estimate of the $L_{IF}$ PCOs. The result of the LEIAR antenna is equal to the IGS ANTEX $L_{IF}$ PCO of 167 mm. In the case of the TRM5 antenna, the $L_{IF}$ PCO according to the ANTEX entries is 80 mm. Compared to the manual PCV applied result of 72 mm, a bias of 8 mm persists, which is consistent with the result on the second row. Except for the 8 mm difference in the TRM5 due to using data from different days, the agreement of the $L_{IF}$ PCOs indicates the successful implementation of the manual PCV correction to the input RINEX observations.

**Figure 5:**
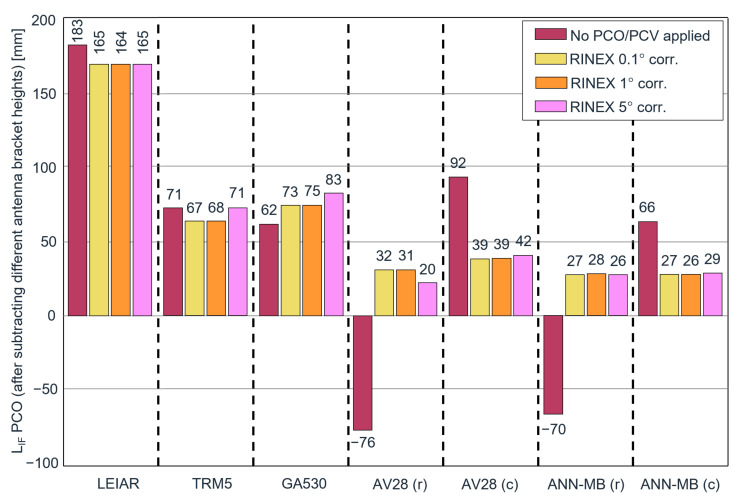
PPP height differences regarding the reference height before and after applying the PCV corrections. The abbreviations (c) and (r) in the antenna names correspond to the utilized mounting brackets (circular plane and rectangular bracket).

Since no official calibrations are freely available for the remaining antennas, they could not be compared against an absolute reference. However, by accounting for the different adaptor heights, the height estimations can be compared. Figure 5 shows the height offsets from the GPS pillar reference point using the original data without supplying the antenna type in the RINEX header and after correcting the RINEX data with the estimated PCV corrections in 0.1°, 1°, and 5° resolution. Since we compare with a known height and do not apply corrections for the PCO, the observed height offsets can be interpreted as an $L_{IF}$ PCO estimate.

The first aspect to note is the height difference with and without applying receiver antenna PCV corrections. For the LEIAR antenna, the difference is 18 mm, whilst the TRM5 antenna demonstrates almost no changes (between 0 and 4 mm) with or without the PCV correction. It means that the phase variations from the TRM5 antenna must be close to a circular constant phase pattern, while the LEIAR and other antennas apparently deviate more from a circular pattern, which eventually results in the observed height offset. It is known that radomes, such as the one used by the LEIAR, can have an impact on the station height of several cm (see also [20]). Moreover, the effect of applying PCV corrections is much higher for both lower-cost antennas (AV28, ANN-MB) than for the more expensive antennas (LEIAR, TRM5, and GA530). The lower-cost antennas AV28 and ANN-MB are characterized by high offsets of 92 and 66 mm before applying the PCV corrections. After applying the corrections, the estimated $L_{IF}$ PCOs are reduced to 26 and 39 mm, which appear rather reasonable. Interestingly, when a rectangular adaptor is utilized, the offsets are of opposite sign but in the same order of magnitude of approximately −70 mm before calibration. After calibration, they are reduced to around the same level as the results with a circular ground plane with offsets of 31 and 28 mm. In particular, the fact that the offsets with the same antenna but with different mounting adaptors differ between 17 and 14 cm before correction and only up to 1 cm after the correction appears promising.

The results for various resolution bins (averaging width) do not vary significantly, with values between 1 and up to 4 mm, except for the GA530 antenna and AV28 antenna with a rectangular adaptor in combination with a bin size of 5°. The latter could be due to a measurement outlier or caused by substantial PCV differences, presumably caused by the near-field effects on the edges of the rectangular adaptor, which might be better represented at the finer scale but may behave poorly in the case of the 5° averaging method.

### 6.2. RTKLIB PPK Results

**Figure 6:**
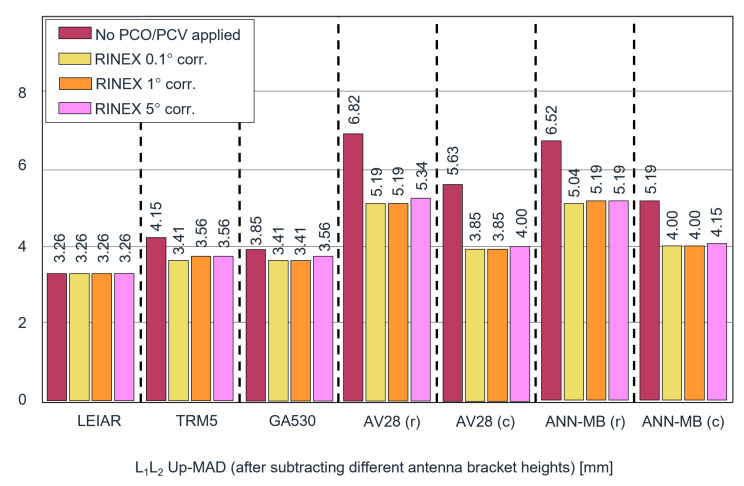
RTKLIB PPK MADs of the Up component of the kinematic positions before and after input data correction. The abbreviations (c) and (r) in the antenna names correspond to the utilized mounting brackets (circular plane and rectangular bracket).

**Figure A3:**
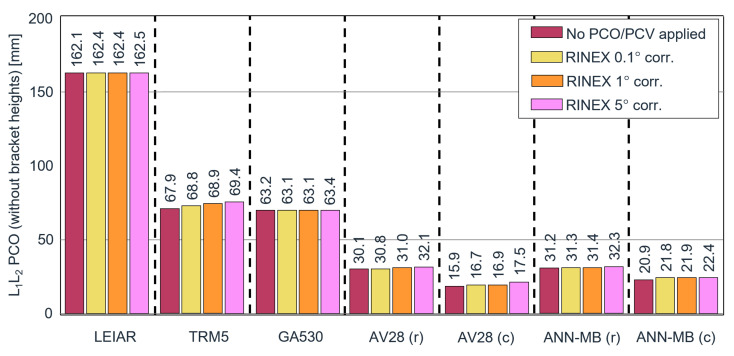
PPK estimated $L_{1}$ + $L_{2}$ Up-component offsets. The abbreviations (c) and (r) in the antenna names correspond to the utilized mounting brackets (circular plane and rectangular bracket).

Post Processed Kinematic (PPK) positioning results, with and without antenna PCV corrections, are obtained using RTKLIB. The correction is performed by modifying both base and rover RINEX datasets with the absolute PCVs. However, the same effect can be achieved by correcting the rover data with the mean of the stacked SD residuals from which the absolute PCV values are derived. We provide results after correcting the RINEX files according to the different PCV averaging widths obtained from the calibration procedure (0.1°, 1°, and 5°). One full day is processed for each antenna and only ambiguity-fixed estimations are used. As with the calibration step, the IGS station DLF1 is used as the base station. Errors caused by the troposphere or ionosphere are assumed equal and eliminated in the process. Other parameters, i.e., positioning components and clock errors, are estimated in the Kalman filter as parameters. One of the main goals of antenna calibration is to increase the repeatability of kinematic position solutions. We therefore analyze the positioning performance by computing the MAD of the kinematic North, East, and Up (NEU) components, with the results for the vertical component given in Figure 6. For each run, also the median offset in the NEU components is reported, using the $L_{1}$ + $L_{2}$ RTKLIB solution with results for the vertical component given in Figure A3. These provide estimates of the “$L_{1}$ + $L_{2}$” PCO. Processing in RTKLIB can be done with $L_{1}$ only, or $L_{1}$ + $L_{2}$ combined. To use all observations, the $L_{1}$ + $L_{2}$ option is used, but this results in a single $L_{1}$ + $L_{2}$ position estimate (likewise, for the calibration itself, $L_{1}$ + $L_{2}$ processing is used to obtain residuals for both $L_{1}$ and $L_{2}$). In the ANTEX files, only separate $L_{1}$ and $L_{2}$ PCOs are available, and the combined PCO for $L_{1}$ + $L_{2}$, which depends on the weighting applied in RTKLIB, is not available. However, this does not matter here, as, in our calibration procedure, the PCVs are given with respect to the combined $L_{1}$ + $L_{2}$ PCO, and there is no reason that the $L_{1}$ and $L_{2}$ PCO cannot be the same as long as it is consistent with the provided PCV for each frequency. Another useful observation to make here is that the positioning results are not sensitive to a constant shift in the PCV: any constant that is added to the PCV is absorbed by the clock estimate in the positioning estimate. This means that we can have PCO/PCV calibrations for which not only the $L_{1}$ and $L_{2}$ PCO is the same, but also the $L_{1}$ and $L_{2}$ PCV at zenith is zero, as is the case in our calibrations.

The MAD for the kinematic Up component from the $L_{1}$ + $L_{2}$ solution is illustrated in Figure 6. Results containing the horizontal components and $L_{1}$-only results can be found in Table A2–Table A4 in the Appendix A.

For the results without antenna phase center corrections, the highest-cost antennas (LEIAR, TRM5, and GA530) have the smallest vertical MADs. They vary around 3 to 4 mm. This is rather unsurprising for the LEIAR antenna because the phase effects largely cancel out over the short baseline, yet similar performance is found for the TRM5 and GA530 antennas. The AV28 and ANN-MB antennas yield 5.6 and 5.2 mm, respectively. More importantly, after applying the corrections to the data, the precision always increased (or remained equal as in the reference LEIAR case). After correcting, the MAD for the higher-cost TRM5 and GA530 antennas was reduced to 3.6 and 3.4 mm, approaching the LEIAR result (3.3 mm). The MADs of the AV28 and ANN-MB antennas were lowered significantly by 31% (3.9 mm) and 23% (4 mm), respectively. This is particularly promising since it brings them to an almost comparable level to the more expensive antennas.

The two cases with a rectangular adaptor showed the highest deviations but also the highest improvements after calibration, from 6.8 and 6.5 mm MAD down to 5.2 mm. Though the performance is not equal to the corrected results with a circular ground plane, they are on the same level as the uncorrected circular ground plane results and demonstrate the potential to significantly lower the multipath effects by using the proposed method. The observed effects are presumably caused by the reflections in the near field from the metallic rectangular adaptor.

The corrections with 5° binning size perform slightly worse than the higher-resolution ones. However, only minor differences are visible between the 0.1° and 1° binning size results. It implies that the highest resolution of 0.1° is not necessary and that the 1° binning size appears to be a good trade-off between additional computing time requirements and smoothing potential outliers in the observations.

Investigating the estimated PCOs from the RTKLIB processing using $L_{1}$ + $L_{2}$ data reveals consistency, even when no PCV is applied. This is quite understandable as our PCVs are computed from residuals from another RTKLIB processing, and the residuals are with respect to the $L_{1}$ + $L_{2}$ position estimate using only a calibration for the base antenna. The median PCO height estimations using $L_{1}$ + $L_{2}$ observations, before and after correcting for the PCVs, are illustrated in Figure A3.

## 7. Conclusions

In this work, we introduce a cost-efficient antenna calibration method that improves the performance of low-cost GNSS receiver antennas up to the level of traditional, high-end GNSS equipment. The calibration procedure can be applied directly in the field and is made available online as a web service (https://gnss-antcal.citg.tudelft.nl, accessed on 9 February 2022). This paves the way for PCO and PCV estimation for high-precision positioning and meteorological and other applications with low-cost equipment.

The quality and remaining residuals in the antenna calibration procedure are assessed and positioning performance is demonstrated in PPP and PPK applications with five antennas of different quality and price.

The analysis of the ambiguity-fixed phase residuals before and after the conducted calibration demonstrates a clear reduction in the MAD for all antennas. Comparing the residuals before calibration to the results of our reference antenna LEIAR25.R3 LEIT, the lower-priced antenna, AV28, was, on average, off by 0.74 mm (29%) on $L_{1}$ and 1.78 mm (63%) on $L_{2}$. For ANN-MB, these values are 1.33 mm (53%) on $L_{1}$ and 2.52 mm (89%) on $L_{2}$. After calibration, the AV28 residuals could be reduced to a difference of 0.3 mm (12%) on $L_{1}$ and 0.44 mm (16%) on $L_{2}$. For ANN-MB, these values were 0.89 mm (35%) on $L_{1}$ and 1.48 mm (52%) on $L_{2}$. In comparison, the higher-grade antennas, TRM5 and GA530, were off by 0.74 mm (29%) and 0.59 mm (23%) on $L_{1}$ and 1.03 mm (36%) on $L_{2}$ before application of the calibration procedure and could be reduced to the same level of the reference antenna on $L_{1}$ and close to 0.29 mm (10%) on $L_{2}$.

With the implemented elevation-based residual averaging method, the phase pattern could be captured for a great part for all antennas. However, some distinctions can be made. It occurs that the TRM5 and GA530 are characterized by an almost circular phase pattern over the full horizon, whilst the lower-priced antennas, AV28 and ANN-MB, indicate a greater azimuthal dependency.

The calibration efficiency of all antennas is demonstrated by evaluating the positioning stability and offsets. First, we investigated the PCV influence and estimated the positioning offsets on the ionosphere-free linear combination phase center in PPP. A height offset of 92 and 66 mm was observed for the lower-priced antennas, AV28 and ANN-MB, before calibration. After calibration, the offsets were reduced to approximately 40 and 27 mm, respectively. Interestingly, the same antennas on a rectangular adaptor that yielded height offsets of −76 and −70 mm before calibration were transformed to similar positive offsets of approximately 30 and 27 mm after calibration. Second, the kinematic positioning stability was analyzed over a short baseline in PPK. Similar to the residual performance, we observed the best positioning stability performance for the highest-quality antennas. Comparably, the lower-priced antennas demonstrated higher variance but improved significantly after the calibration. The MADs in the vertical direction of the low-cost antennas were reduced by 20.4–23.9%. For the AV28 antenna, they were lowered from 5.6 to 3.8 mm. For ANN-MB-00, they were brought from 5.2 to 4 mm. It brings them to a level close to the performance of the antennas LEIAR, TRM5, and GA530, which are characterized by MADs of 3.3, 4.1, and 3.8 mm before and 3.3, 3.6, and 3.4 mm after calibration.

The special case scenario consisting of the antennas AV28 and ANN-MB with a metallic rectangular adaptor demonstrated the highest deviations, the strongest azimuthal dependency, but also the greatest improvements in all cases.

Calibrations can be provided in the internationally accepted IGS ANTEX standard, or directly applied as corrections to RINEX files in the event that the chosen processing software does not support, or only partly supports, the ANTEX format. Examining different binning widths of 0.1°, 1°, and 5° for the elevation-based averaging method revealed, in some cases, a slight performance decrease for the standard 5° resolution over the 1° and 0.1° binning widths. Given the presence of the higher amount of potential outliers in the 0.1° and the increased computational costs, the 1° resolution appears to be a good trade-off between effectiveness and smoothing.

The reported PCOs of the PPK process (Figure A3) are given with respect to the combined $L_{1}$ + $L_{2}$ PCO and are close to the $L_{1}$ PCO. They are consistent with using no PCV corrections, while the estimated PCVs are with respect to the same phase center as obtained without calibration. The presented PCO estimations are subject to improvement due to uncertainty introduced by the manual height measurement. However, even without respecting the PCO, the estimated PCVs are strongly beneficial for deformation or atmosphere monitoring. Further testing using multiple frequencies could widen the application from the current GPS-only to all GNSS frequencies. The provided PCVs and PCOs and the online service to perform the antenna calibrations in the field can be helpful for the widespread application of low-cost antenna and receiver setups for high-precision applications.

## Figures and Tables

**Table 1:** Utilized base and rover receiver and antennas at the experimental site.

Type	Receiver	Antenna
base	Trimble NetR9	Leica AR25.R3 LEIT (LEIAR)
rover	u-blox ZED-F9P	u-blox ANN-MB-00 (ANN-MB) using rectangular adaptor bracket (r)
		u-blox ANN-MB-00 (ANN-MB) using circular plane (c)
		Trimble AV28 (AV28) using rectangular adaptor bracket (r)
		Trimble AV28 (AV28) using circular plane (c)
		Trimble GA530 (GA530)
		Trimble TRM55971.00 NONE, Zephyr 2 geodetic (TRM5)
		Leica AR25.R3 LEIT (LEIAR)

**Table 2:** Height differences for three different scenarios of handling antenna calibrations. The reference is the estimated height for the LEIAR antenna, with IGS ANTEX corrections (5° angular resolution) applied automatically by the PPP. See the text for a more detailed explanation.

Antenna/Case	LEIAR	TRM5
No antenna PCO/PCVs [mm]	183	71
Automatic IGS PCO+PCV [mm]	0	−8
Manual IGS PCV-only [mm]	167	72

**Figure A1:**
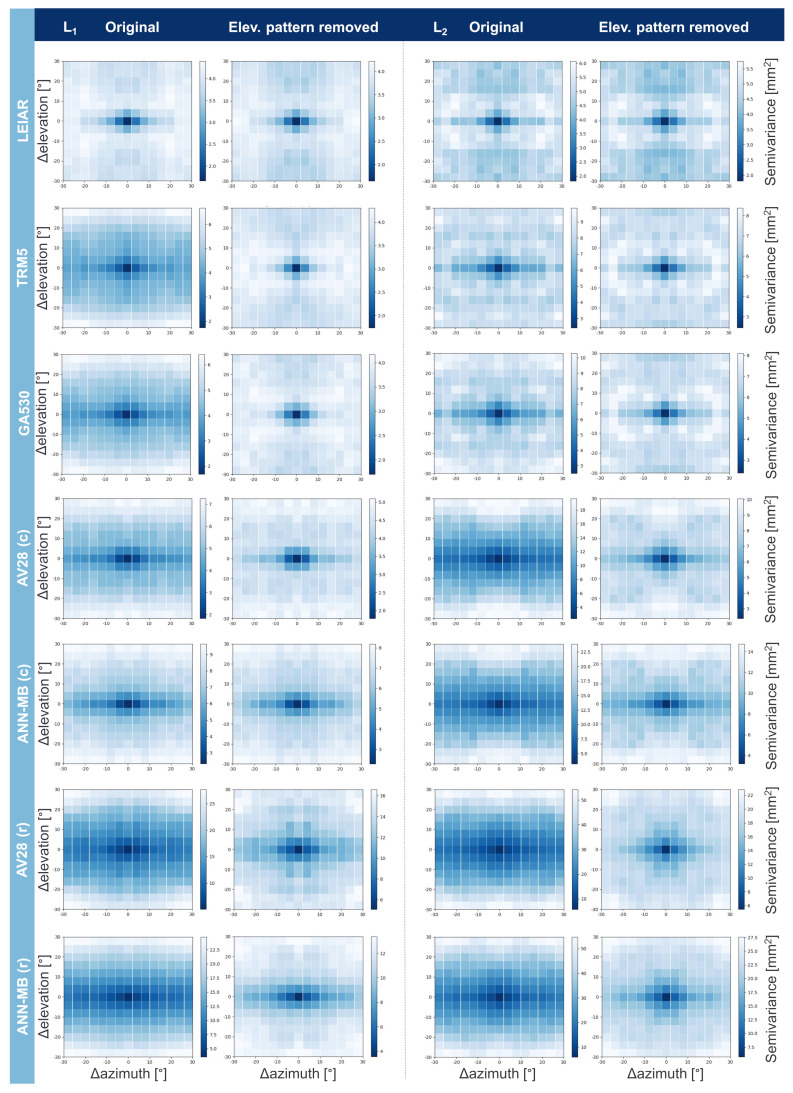
Semivariogram maps of the residuals of all investigated antennas on $L_{1}$ (**left**) and $L_{2}$ (**right**). The figure on the right of each frequency illustrates the residuals after removing the elevation pattern (column elev. pattern removed) from the data. The abbreviations (c) and (r) in the antenna names correspond to the utilized mounting brackets (circular plane and rectangular bracket). Each pixel represents 4° in azimuth and elevation totaling a range of −30° to +30°.

**Figure A2:**
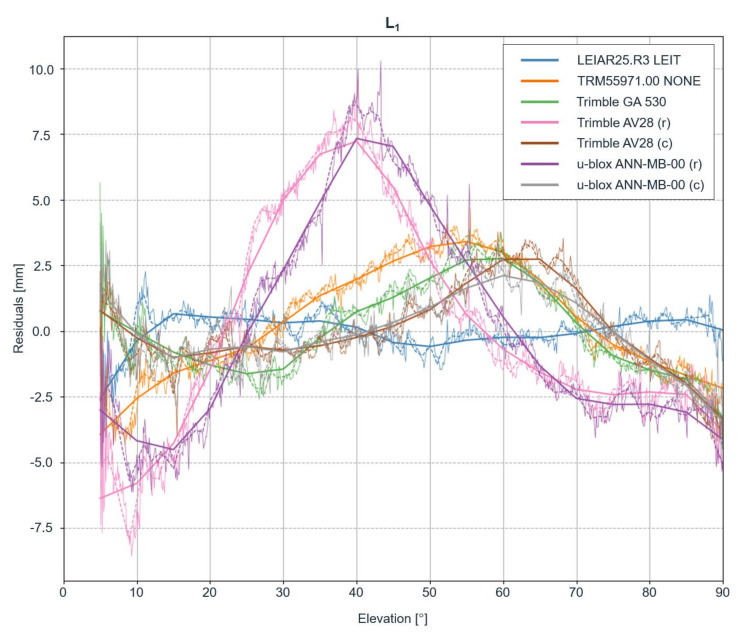

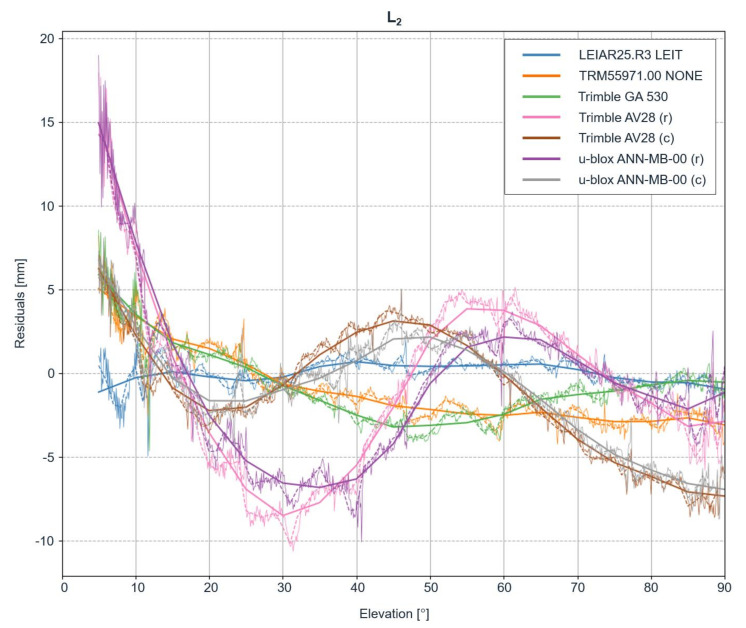
Elevation-dependent residuals on $L_{1}$ (**top**) and $L_{2}$ (**bottom**). The thin lines show the residuals obtained with a 0.1° averaging bin size, the thin dashed lines with 1° averaging bin size, and the thick lines the 5° averages. The terms (r) and (c) correspond to the utilized mounting brackets (circular plane and rectangular bracket).

## Data Availability

Data are made available via the TU Delft/4TU data repository: https://doi.org/10.4121/14743014 (accessed on 9 February 2022).

## Abbreviations

The following abbreviations are used in this manuscript:

ANN-MB	u-blox ANN-MB-00
ANTEX	Antenna Exchange Format
APC	Antenna Phase Center
ARP	Antenna Reference Point
AV28	Trimble AV28
CORS	Continuously Operating Reference Station
DD	Double Difference
DLF1	IGS station DLF1 at NMi building in Delft, Netherlands
DOMES	Directory of MERIT Sites
DPGA	Dutch Permanent GNSS Array
GA530	Trimble GA530
GNSS	Global Navigation Satellite System
GPS	Global Positioning System
IGb14	IGS GNSS geodetic reference IGb14
IGS	International GNSS Service
$L_{1}$	Center signal frequency at 1575.42 MHz
$L_{2}$	Center signal frequency at 1227.60 MHz
$L_{IF}$	Ionosphere-free combination using L1 and L2
LEIAR	Leica AR25.R3 LEIT
MAD	Median Absolute Deviation
MERIT	Monitor Earth Rotation and Intercompare the Techniques of Observation and Analysis
NEU	North, East, Up
NMi	Netherlands Metrology Institute
NRCan	Natural Resources Canada
PCO	Phase Center Offset
PCV	Phase Center Variation
PPK	Post Processed Kinematic
PPP	Precise Point Positioning
RINEX	Receiver Independent Exchange Format
SD	Single Difference
TRM5	Trimble TRM55971.00 NONE
VRS	Virtual Reference Station

## Appendix A

This appendix contains additional information about the processing options, kinematic positioning results of the North, East, and Up components, and diagrams of the semivariances and elevation-dependent patterns. The processing options to obtain the fixed phase residuals in RTKLIB are shown in Table A1. Table A2, Table A3, Table A4 contain the Post Processed Kinematic Median Absolute Deviations (MADs) in North (A2), East (A3), and Up (A4) directions. Each contains results with no corrections applied and the reductions after applying the elevation-dependent calibrations using observations from both frequencies ($L_{1}L_{2}$) and using $L_{1}$-only observations. The semivariances of the fixed phase residuals on $L_{1}$ and $L_{2}$ of all antennas before and after removing the elevation-dependent antenna patterns are illustrated in Figure A1. The elevation-dependent antenna patterns are shown in Figure A2. PCOs of the RTKLIB PPK processing with and without applying PCVs can be found in Figure A3.

**Table A1:** RTKLIB (RNX2RTKP) processing options.

Option	Parameter
Positioning solution	static (-p 3)
Elevation cut-off	5${}^{\circ }$ (-m 5)
Ambiguity Resolution (AR)	fix-and-hold (-h)
AR validation threshold	3 (-v 3.0)
Output positioning	East, North, Up baseline (-a)
Statistics output	residuals (-y 2)
Satellite system	GPS-only (-sys G)
Time format	YYYY/MM/DD hh:mm:ss (-t)
Kalman filter	forward + backward (-c)

**Table A2:** RTKLIB PPK North component MADs in mm using $L_{1}$ and $L_{2}$ data ($L_{1}L_{2}$ column) and $L_{1}$-only observations ($L_{1}$-only column). The uncorrected results (no corr.) are shown on the left. The columns on the right (Δ corrected) show the MAD decrease (indicated by a ‘-’ sign) after applying the elevation-only-based calibrations with the averaging resolutions 0.1°, 1°, and 5°.

	MAD [mm]							
	$L_{1}L_{2}$				$L_{1}$-only			
	no corr.	Δ corrected			no corr.	Δ corrected		
		0.1${}^{\circ }$	1${}^{\circ }$	5${}^{\circ }$		0.1${}^{\circ }$	1${}^{\circ }$	5${}^{\circ }$
LEIAR25.R3 LEIT	1.9	±0.0	±0.0	±0.0	2.2	±0.0	±0.0	±0.0
TRM55971.00 NONE	2.2	−0.3	−0.1	−0.1	2.7	−0.6	−0.6	−0.6
Trimble GA530	2.2	−0.1	−0.1	−0.1	2.7	−0.4	−0.4	−0.4
Trimble AV28(rectangular bracket)	4.2	−0.9	−0.9	−0.7	4.6	−0.7	−0.7	−0.7
Trimble AV28(circular plane)	2.7	−0.6	−0.6	−0.6	2.4	−0.1	±0.0	±0.0
U-blox ANN-MB-00(rectangular bracket)	4.7	−1.2	−1.2	−1.2	4.7	−0.9	−0.9	−0.9
U-blox ANN-MB-00(circular plane)	3.0	−0.3	−0.3	−0.3	3.0	−0.2	−0.2	−0.2

**Table A3:** RTKLIB PPK East component MADs in mm using $L_{1}$ and $L_{2}$ data ($L_{1}L_{2}$ column) and $L_{1}$-only observations ($L_{1}$-only column). The uncorrected results (no corr.) are shown on the left. The columns on the right (Δ corrected) show the MAD decrease (indicated by a ‘-’ sign) after applying the elevation-only based calibrations with the averaging resolutions 0.1°, 1°, and 5°.

	MAD [mm]							
	$L_{1}L_{2}$				$L_{1}$-only			
	no corr.	Δ corrected			no corr.	Δ corrected		
		0.1${}^{\circ }$	1${}^{\circ }$	5${}^{\circ }$		0.1${}^{\circ }$	1${}^{\circ }$	5${}^{\circ }$
LEIAR25.R3 LEIT	1.3	±0.0	±0.0	±0.0	1.6	−0.1	±0.0	±0.0
TRM55971.00 NONE	1.5	±0.0	±0.0	±0.0	1.8	−0.2	−0.2	−0.2
Trimble GA530	1.5	−0.1	−0.1	−0.1	1.8	−0.2	−0.2	−0.2
Trimble AV28(rectangular bracket)	2.8	−0.7	−0.6	−0.6	3.9	−1.3	−1.3	−1.2
Trimble AV28(circular plane)	1.8	−0.2	−0.2	−0.2	1.8	−0.2	±0.0	±0.0
U-blox ANN-MB-00(rectangular bracket)	3.4	−1.0	−1.0	−0.9	4.3	−1.8	−1.8	−1.6
U-blox ANN-MB-00(circular plane)	2.4	−0.3	−0.3	−0.3	1.9	±0.0	±0.0	±0.0

**Table A4:** RTKLIB PPK Up component MADs in mm using $L_{1}$ and $L_{2}$ data ($L_{1}L_{2}$ column) and $L_{1}$-only observations ($L_{1}$-only column). The uncorrected results (no corr.) are shown on the left. The columns on the right (Δ corrected) show the MAD decrease (indicated by a ‘-’ sign) after applying the elevation-only based calibrations with the averaging resolutions 0.1°, 1°, and 5°.

	MAD [mm]							
	$L_{1}L_{2}$				$L_{1}$-only			
	no corr.	Δ corrected			no corr.	Δ corrected		
		0.1${}^{\circ }$	1${}^{\circ }$	5${}^{\circ }$		0.1${}^{\circ }$	1${}^{\circ }$	5${}^{\circ }$
LEIAR25.R3 LEIT	3.3	±0.0	±0.0	±0.0	3.6	±0.0	±0.0	±0.0
TRM55971.00 NONE	4.2	−0.7	−0.6	−0.6	4.7	−1.0	−1.0	−0.9
Trimble GA530	3.9	−0.4	−0.4	−0.3	4.6	−0.7	−0.7	−0.7
Trimble AV28(rectangular bracket)	6.8	−1.6	−1.6	−1.5	7.4	−1.3	−1.2	−1.2
Trimble AV28(circular plane)	5.6	−1.8	−1.8	−1.6	5.3	−1.2	−1.2	−1.2
U-blox ANN-MB-00(rectangular bracket)	6.5	−1.5	−1.3	−1.3	7.3	−1.8	−1.8	−1.6
U-blox ANN-MB-00(circular plane)	5.2	−1.2	−1.2	−1.0	5.6	−0.7	−0.7	−0.7
